# The Theoretical Construction of a Classification of Clinical Somatic Symptoms in Psychosomatic Medicine Theory

**DOI:** 10.1371/journal.pone.0161222

**Published:** 2016-08-15

**Authors:** Fanmin Zeng, Xueli Sun, Bangxiang Yang, Hong Shen, Ling Liu

**Affiliations:** 1 College of Sociology and Psychology in Southwest University for Nationalities, Chengdu, Sichuan, People’s Republic of China; 2 Department of Psychiatry, West China Hospital, Sichuan University, Chengdu, Sichuan, People’s Republic of China; 3 Department of Pain Management, West China Hospital, Sichuan University, Chengdu, Sichuan, People’s Republic of China; 4 Department of Urology, West China Hospital, Sichuan University, Chengdu, Sichuan, People’s Republic of China; 5 Department of Digestive Internal Medicine, West China Hospital, Sichuan University, Chengdu, Sichuan, People’s Republic of China; Sichuan University, CHINA

## Abstract

**Objective:**

This article adopts the perspective of psychosomatic medicine to present and test a theoretical model of the classification of clinical somatic symptoms. The theoretical model consists of four dimensions: emotional somatic symptoms, biological somatic symptoms, imaginative somatic symptoms, and cognitive somatic symptoms.

**Method:**

A clinical somatic symptom classification scale was developed according to the theoretical model. A total of 542 participants completed the clinical somatic symptoms classification scale. The data were analyzed using exploratory and confirmatory factor analyses.

**Results:**

The results confirmed the theoretical model. The analyses found that the proposed theoretical structure of the scale was good, as indicated by factor loadings and fit indices, and that the scale had good reliability and construct validity.

**Conclusions:**

Based on the interpretation of the clinical symptoms of psychosomatic medicine, the treatment of chronic non-infectious diseases includes at least three dimensions: the first is the etiological treatment, the second is the pathophysiological and pathopsychological dimension, and the third is symptomatic treatment. The unified psychosomatic point of view and diverse clinical thinking modes are aimed at identifying different classes of somatic symptoms and important prerequisites for the treatment of these symptoms. We registered the study with the Chinese Clinical Trial Registry and it was approved by the West China Hospital, Sichuan University ethics committee. Trial registration: The registration number is ChiCTR-OCS-14004632 (time: 2014-05-12).

## Introduction

Form this point of view clinical disciplines must address the various somatic symptoms of patients during consultation. According to conventional clinical thought, symptoms always have a corresponding pathological basis [1]. Thus, somatic symptoms offer insight into the various pathological changes in the body and can serve as a starting point, or a fundamental basis, for diagnosis and treatment. These symptoms are classified according to organ systems, for example as digestive symptoms, respiratory symptoms, cardiovascular symptoms, or nervous system symptoms. This approach helps a clinician decipher the symptoms’ clues and then manage the case through triage, examination, and a treatment plan.

However, there are several problems associated with this traditional way of thinking. First, symptoms corresponding to a system of organs do not necessarily indicate that the particular system has problems. Therefore, if analysis of the symptoms points to a system and no problems are found, the diagnosis and treatment cannot proceed. If the patient is referred to other institutions, this means a new diagnosis and treatment, new expenditure of diagnostic resources, and a waste of time. Some symptoms overlap in the classification of diseases; thus, it can be difficult to pinpoint the affected organ system. For example, vomiting can be classified as a digestive system symptom and also as a nervous system symptom. A cause will lead to an effect, and this mode of universal thinking can dominate clinical thinking, which can become rigid and standardized. The fact is that a single cause may not necessarily lead to a specific effect, whereas an effect can be a result of many causes. The lack of diversity in thinking and the failure to consider a problem from multiple perspectives, out of force of habit, will lead to rigid thinking in clinical and research work and will have a negative effect on understanding the disease and treatment.

The objectives of this paper are as follows: (1) to put forth a theoretical model of classification of clinical somatic symptoms in psychosomatic medicine; (2) to use the psychological assessment method to test and verify the applicability and scientific validity of the theoretical model for classifying clinical somatic symptoms in psychosomatic medicine; (3) to draw on modern psychometric methods to develop a diagnostic instrument (a rating scale) based on the classification of clinical somatic symptoms for the practical application of this theory; and (4) to show that the proposed theory can support a set of stable and effective scientific evaluation tools.

## Materials and Methods

### Theoretical model of the classification of clinical somatic symptoms within the framework of psychosomatic medicine

Clinical classification and diagnosis are intended to target treatment and to generate accurate ideas for clinical diagnosis and treatment. As mentioned above, adherence to the conventional system that is designed to decipher somatic symptoms in the traditional way can lead to drawbacks and can lead to misconceptions. For example, the International Association for the Study of Pain in 1979 defined pain as “an unpleasant sensory and emotional experience associated with actual or potential tissue damage or described in terms of such damage” [2]. This definition stresses the importance of the emotional and suffering aspects of clinical somatic symptoms. As recognized and understood according to the above definition, a physical symptom is essentially a “subjective feeling,” and this “feeling” is related to “damage” or “potential damage”. In addition, physical symptoms are associated with the subjective experience of the individual. In other words, a physical symptom may not have a purely biological source, but it is always related to cognition, emotion, personality, and other psychological elements [3]. Based on this idea, somatic symptoms can be defined in additional ways. First, these symptoms can be viewed as organic reactions to an emotion. This definition comes from the theory of alexithymia [4]. Alexithymia [4] is a personality construct that denotes a deficit in the cognitive processing and regulation of emotion. This deficit may, in turn, result in a negative affect state that fosters a hypervigilance toward somatic sensations and increased report of somatic complaints. Organ function changes in response to demands in the biology represent a universal phenomenon, such as the often-mentioned example where the lower animals in biological evolution animal appears because of fear of urinary incontinence or muscle tremors; in daily life, "I'm sick of the sight of you," and so is the change in organ function. The evolution and development of the human nervous system, in which the main form of human expression is verbal or emotional, is such that some individuals will show changes in organ function as an expression of "the main way to demand"; this response has been termed "alexithymia".

Second, somatic symptoms represent an important way to relieve inner conflict. This definition comes from psychoanalytic theory [5], specifically the psychological defense mechanism Rationalization. Freud believed that somatic symptoms were the expression of a defense mechanism and that somatic symptoms were a way to ease inner conflict. Third, somatic symptoms can be viewed as the emotion itself, specifically “an unpleasant subjective experience”, mainly related to anxiety and depression. The clinical medicine interpretation of anxiety is as the inner experience of anxiety or fear associated with an autonomic nervous system function disorder and motor restlessness. Anxiety, therefore, relates to the bodily level, experience level and cognitive level [3]. Fourth, symptoms can be views as negative interpretations by the individual of a feeling. This definition mainly implies that the cognitive system plays a major role in people with somatic symptoms. Various feelings exist at any time, through cognitive effects, and if the individual has a positive interpretation of a feeling, then the individual now has a need to feel. However, if that feeling is negative, then this feeling will become a somatic symptom [6]. Lastly, somatic symptoms can be viewed as the result of learning or imitation. This definition is mainly seen in suggestion or autosuggestion circumstances, where individuals can reproduce the symptoms or copy the previous symptoms [7].

According to the above descriptions of somatic symptoms, patients, even those with pathological changes to the body have two components of somatic symptoms: one is the “biological component”, while the other is the “psychological component”. Using the theory of psychosomatic medicine to interpret clinical somatic symptoms shows that the problem is guiding diversified thinking in clinical work. Inertia thinking on medicine is a unitary mode of thinking. If clinicians always proceed only from the angle of pathological damage to find the reason for the symptom and they give the corresponding treatment, while ignoring the “psychological component”; thus, patients with somatic symptoms will not have their symptoms resolved satisfactorily. It is worth noting that somatic symptoms incorporate the mentality of the patients, including their quality of life, treatment compliance and prognosis. In addition, the law of sufficient reason suggests [8] that there should exist an inevitable logical link between premises and conclusions. Symptomatic cases can exist, at the same time as the damage, thus judging case symptoms as being only caused by damage is in violation of the law of sufficient reason, which in the process of thinking, it is necessary to make sure that a judgment or an argument is true and not false. If there is a lack of reason, there is no argument. The psychosomatic medical interpretation of somatic symptoms provides a clinical basis for a comprehensive analysis of somatic symptoms and analyses of different individuals with the same symptoms. Then, according to the viewpoint of psychosomatic medicine in interpreting somatic symptoms, there are at least three approaches to the treatment of chronic non-infectious diseases: the first is the etiological treatment latitude, the second is the pathological, physiological, and pathological psychotherapy latitude, and the third is the symptom treatment latitude. Therefore, greater attention should be paid to the independent treatment of somatic symptoms.

On the basis of careful analysis, this paper presents a comprehensive evaluation from the standpoint of psychosomatic medicine [9] (The theoretical framework is centered on the bio-psycho-social model). Accordingly, somatic symptoms can be roughly divided into the following four categories.

### Biological somatic symptoms

These are mainly produced by physical, chemical, and biological factors that are a direct result of partial damage to nerve endings or the result of local tissue injury after biochemical reactions caused by the suboptimal stimulation of nerve endings.

### Emotional somatic symptoms

In this case, the somatic experience is rooted in negative emotional symptoms. According to the theory of alexithymia [4], a negative affect state will foster a hypervigilance toward somatic sensations and increased report of somatic complaints. So, somatic symptoms are organic reactions to the emotion. In general, the reaction is from a negative emotion, especially depression and anxiety.

### Imaginative somatic symptoms

This category refers to patient symptoms that result from imagination, suggestion, or autosuggestion. The typical characteristics of these symptoms are variety and “over-constancy.” For instance, some patients report feeling two matches stuck in the lumen of their throat, which prevents them from swallowing freely and leads to pain during swallowing.

### Cognitive somatic symptoms

The word cognitive here has two meanings. The first refers to the individual interpretation of body awareness. Many types of somatic feelings are always present, but it is only when an individual has a negative interpretation of some feeling at the cognitive level that this feeling may become a somatic symptom. An illusion in psychiatry is defined as a situation in which there is no objective stimulus, but stimulation of the sensory nerves in an organ is present, causing the perceptual experience corresponding to the organ [10]. The second meaning of cognitive somatic symptoms is that some physical symptoms match the definition of an illusion. For example, bilateral tinnitus can be diagnosed and treated as an illusion. The characteristics of cognitive somatic symptoms are relatively fixed in their characteristics and location, and the symptoms are clear and vivid.

### A study of the clinical somatic symptoms scale

#### Objective

An empirical study was conducted to validate the proposed theoretical model

#### Participants

The study investigated three samples. **The first sample** included participants who were pain, urology, and digestive internal medicine outpatients and psychiatric patients who visited the West China Hospital of Sichuan University between May 2014 and Sep 2014. The first sample surveyed 232 patients, and the male to female ratio of these cases was 1:3. **The second sample** was a predictive sample that included the questionnaire results of 198 participants who were pain, urology, and digestive internal medicine outpatients and psychiatric patients who visited the West China Hospital of Sichuan University between October 2014 and December 2014. **The third sample** included 542 patients administered the second version of the questionnaire; of these, 292 were randomly selected as samples for exploratory factor analysis (EFA). The remaining 250 questionnaires were used for confirmatory factor analysis (CFA). All participants were pain, urology, cardiology, neurology and digestive internal medicine outpatients and psychiatric patients who visited the West China Hospital of Sichuan University between Jan 2015 and May 2015. The study was approved by the Chinese Trial Registry Board, and written informed consent was obtained from all of the participants. The registration number for this study is ChiCTR-OCS-14004632.

The inclusion criteria included the following: (1) patients between 18 and 65 years old; (2) patients suffering from chronic noninfectious diseases from various branches of medicine; (3) patients with somatic symptoms who expressed concern for pain or affected social functioning; (4) patients with normal sight, hearing and cognitive ability; (5) patients who understood the study’s goals and expressed a desire to cooperate with the investigators; and (6) patients who voluntarily participated in the study.

The exclusion criteria were as follows: (1) patients with an acute infection, acute trauma or disease and perioperative patients with acute-phase disease; (2) patients in critical condition or dying; (3) pregnant or breast-feeding women; (4) patients with substance abuse; and (5) patients who may not comply with treatment, according to the clinical physician’s judgment.

#### Instrument

A survey questionnaire was used to collect the information in the first sample. The questionnaire was developed as follows. First, based on a literature review and clinical experience, the study doctors were asked to list common somatic symptoms, with specific reference items about somatic symptoms contained in the Patient Health Questionnaire (PHQ-15) scale [11], the Symptom Check List (SCL-90) [12], and the Cornell Medical Index (CMI) [13,14]. Second, the somatic symptoms were encoded, summarized and classified. We classified the similar symptoms into a category, for example: fall asleep and wake up early were sleep problems. Then, 124 initial items were incorporated into a questionnaire using a five-point Likert-type scale, which collect the information about the somatic symptoms. All items randomly arranged, five points scoring were very, somewhat, neutral, not very and not at all and recorded as 4,3,2,1,0. Third, five clinical doctors and senior medical graduates inspected and evaluated the readability of the questionnaire and the appropriateness of its scientific content. The final 105 items were randomly distributed throughout the questionnaire to form **the first version of the somatic symptoms checklist**.

#### Procedures

**The first version of the somatic symptoms checklist** was used to survey the second sample group. Survey data were analyzed using principal component analysis. The result of the first Principle Components Analysis, 16 items were deleted because they did not significantly correlate with the total score, and the square of the multiple correlations was less than 0.30. The remaining 89 items were arranged to form **the second version of the questionnaire**, which was used to survey the third sample group. An EFA was performed on the data from 292 patients who completed the second version, from which the structure of the preliminary clinical somatic symptoms was established. CFA was performed on the data for the remaining 250 patients in the third sample group, which established a reasonable assessment of the structure of the clinical somatic symptoms. Data were processed and statistically analyzed using SPSS 19.0 and LISREL 8.72 software (IBM, Chicago, IL, USA).

## Results

### EFA of the classification structure of clinical somatic symptoms

This study analyzed the structure of clinical somatic symptoms using EFA [15]. The second principal component analysis included 18 factors. All of the indices were in accordance with the standard assessment of the appropriateness of factor analysis. The Kaiser Meyer Olkin test value was 0.906, indicating that the sample size was suitable for factor analysis. The Bartlett spherical inspection *χ*^*2*^ value was 2319.954 (*df* = 153, *p <* 0.001), and it reached a significant level. This result indicated that there was a common factor in the overall correlation matrix, further indicating the suitability for factor analysis. According to Principal Component Analysis and the structure of the scree plot, four significant factors were extracted (eigenvalues greater than 1), and the maximum variance orthogonal rotation load value was greater than 0.3, communality was greater than 0.2, and the retention of 55 items explained 61.183% of the total variance.

### Validity testing of the structure of the clinical somatic symptoms classification assessment

#### Content validity

Content validity plays an important role in clinical evaluation [16]. The content validity of the clinical somatic symptoms classification model in this study could largely be evaluated through previous research (such as clinical research, theory formulation, and professional judgment); the questionnaire showed good content validity.

#### Structure validity

EFA and CFA were used to validate the model during the process of developing and testing the instrument derived from theory [17]. The inclusion and exclusion standards were the same as those in study 1. Table 1 shows that the factor loadings (*t* values > 2.00) of each analysis reached the standard level of statistical significance, which showed that four dimensions, or factors, formed an effective index of the construct factors and that the measurement of constructs was appropriate.

**Table 1:** Load factor of the four factors in the scale (LAMBDA-X).

Dimension	Factor 1 emotion	Factor 2 biology	Factor3 imagination	Factor4 cognition
Emotion	0.95 (19.87)			
Sleeping problem	0.69 (12.26)			
Strangeness	0.76 (13.85)			
Pain		0.78 (14.0)		
Limb or trunk weakness		0.82 (15.1)		
Feel cold or fever		0.84 (15.53)		
Disease awareness			0.70 (12.1)	
Suggestibility			0.85 (15.57)	
Shivering				0.86 (16.80)
Chest tightness				0.79 (14.70)
Amnesia				0.88 (17.39)
Buzzing in brain				0.81 (15.23)
Throat sensation				0.62 (10.61)

Note: Figures in brackets for the *t* value.

CFA fit indices for the total clinical somatic symptoms scale (Table 2)

**Table 2:** The fitting index of confirmatory factor analysis of the scale.

Model	χ2	*df*	χ2/*df*	GFI	NNFI	CFI	NFI	RMSEA
M	145.90	59	2.1	0.92	0.98	0.98	0.97	0.07

Table 2 shows that the various indices showed a good fit between the measured data and the assumed model [18].

**Fig 1:**
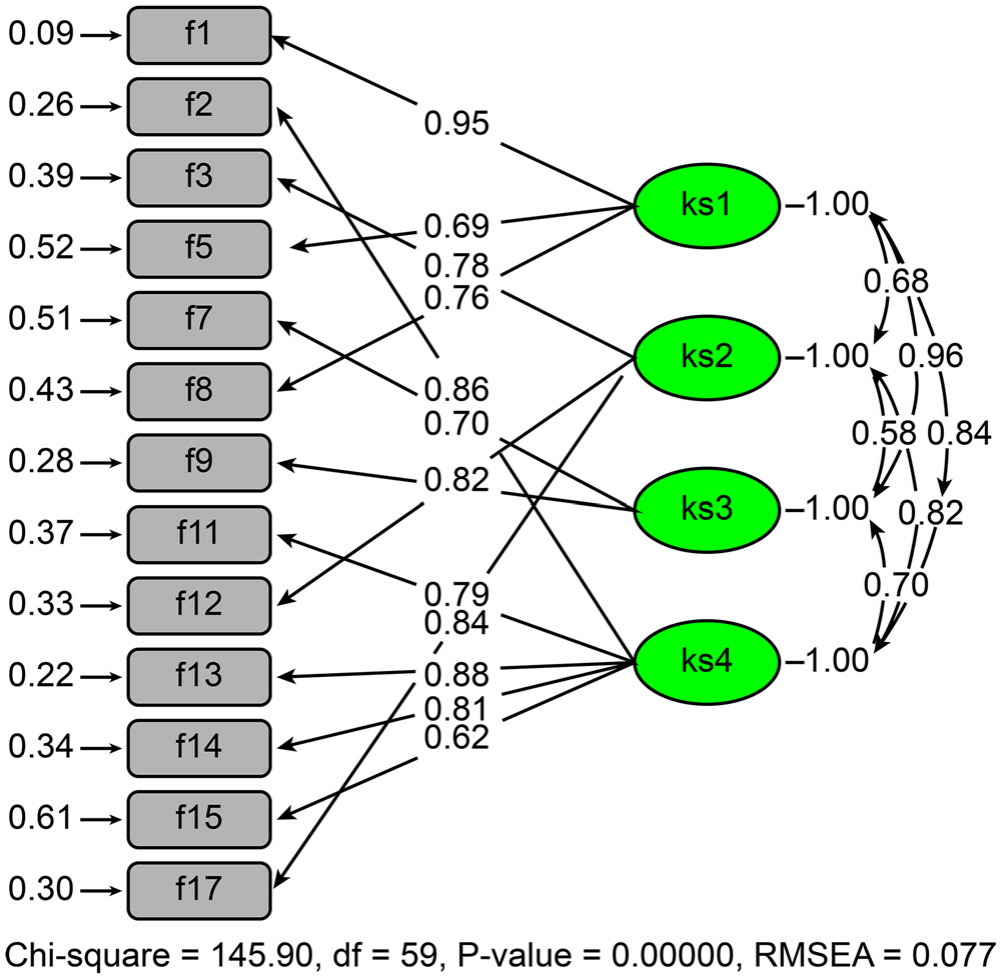
Somatic symptoms in the internal structure model.

Diagram of the internal structure variance model of clinical somatic symptoms (Fig 1)

Fig 1 shows the somatic symptom internal structure model. The factor analysis verified our hypothesis for clinical somatic symptom classification and showed that the questionnaire had fairly good content validity and construct validity.

#### Validity of the test

Two clinicians, each with 5 years of working experience, evaluated independently the participants’ complaints of symptoms, which gave the clinical symptoms assessment result 1 (first result regarding clinical assessment). The clinical symptoms assessment result 2 (second result regarding the clinical somatic symptoms scale) was assessed according to the clinical somatic symptoms scale. The calculated kappa value of result 1 and result 2 was 0.766 (> 0.6). The results showed that the scale has good test validity. The kappa statistic [19] was employed to evaluate concordance between the two methods.

Reliability of the test (Table 3)

**Table 3:** Reliability of the four factors and the total scale.

	α coefficient	Split-half reliability
Factor 1 (emotion)	0.681	0.795
Factor 2 (biology)	0.761	0.806
Factor 3 (imagination)	0.799	0.779
Factor 4 (cognition)	0.825	0.820
Total test	0.824	0.922

Table 3 shows that the questionnaire’s reliability was very good, which indicates that the questionnaire does not need to be modified as a result of these analyses.

## Discussion

This study was based on a summary of clinical experience and an analysis of the factor structure of the classification of symptoms. Somatic symptoms include emotional, biological, imaginative, and cognitive symptoms.

Somatic symptoms can manifest themselves as changes in mood, especially as the direct expression of negative emotions resulting from the perception of somatic symptoms. In such cases, the somatic symptoms cause the emotions themselves. According to Freud’s psychoanalytic theory, individuals can also express emotion through somatic symptoms [5]. For example, in some people with somatic symptoms, the symptoms may replace their emotions when they do not experience acceptance by others or by themselves or when they avoid contact [20]. The alexithymia theory [21] postulates that patients with alexithymia exaggerate the normal feeling in a body part and distort the arousal of somatic symptoms. The patient may often express emotional distress through somatic symptoms. In medical patients, emotional distress may also result from the treatment of their somatic symptoms.) Thus, depressed patients may present with somatic symptoms rather than emotional problems [22]. Biological somatic symptoms are closely related to the pathological changes in the organ or system of organs. Somatic symptoms can also represent images of past experiences or learning as a result of them; the individual’s somatic symptoms derive from imagination, suggestion, or self-talk. For instance, hysterical blindness may involve an important regulation mechanism relevant to this topic. Longitudinal studies have found that children’s persistent abdominal pain is associated with the poor health of their parents [22,23]. Other researchers believe that psychological features are useful for classifying patients with somatic symptoms [24]. The cognitive theory postulates that the body reflects the central nervous system symptoms in peripheral tissues and organs; this concept is necessary for understanding relevant information and somatization related to information processing, such as the understanding of pain. Pain can be verified objectively, and interpretation of nervous system aberrations reveals individual differences. If the feeling of objective existence is interpreted as a negative emotion, this phenomenon can lead to the formation of somatic symptoms. Such individuals show personality traits and an innate genetic susceptibility that lead them to misconstrue signals in the body as physical sensations related to a serious physical illness. It is thought that the above problem results from the lack of cognitive resources to understand bodily symptoms, in which too much focus is placed on the bodily symptoms. However, it is difficult to allocate cognitive resources to attend to the accompanying symptoms and the emotional stress of life events, other aspects of an information-based society, and stressful situations. Thus, the process leads to activation of a symptom and the inappropriate attribution cycle of the somatization of symptoms [6].

The classification of somatic symptoms described above can be applied as follows: (1) to develop clinical thinking and improve understanding of physical symptoms to better implement the unified psychosomatic point of view; (2) to help establish guidelines for the diagnosis of somatic symptoms and mental illness; and (3) to help establish guidelines for the treatment of somatic symptoms, such as the management of weak symptoms with antidepressant treatments and irritable emotional symptoms with anti-anxiety drugs. Cognitive symptoms can be alleviated with cognitive-behavioral therapy, and biological symptoms can be alleviated by means of local and systemic physical treatment. It is worth noting that the methods intended to increase understanding of bodily symptoms from additional perspectives should vary from patient to patient and implement a diverse mode of thinking. Nevertheless, the unified psychosomatic point of view and diverse clinical thinking modes are aimed at identifying somatic symptoms and are important prerequisites for the treatment of these symptoms.

## Conclusion

This paper developed a classification theory of clinical somatic symptoms from the perspective of psychosomatic medicine, which includes emotional, biological, imaginative, and cognitive somatic symptoms. Moreover, the theoretical model for this theory was supported by empirical research. The clinical somatic symptom classification scale showed good reliability, content validity and construct validity and can thus be applied as an evaluation tool. Based on the interpretation of the clinical symptom of psychosomatic medicine, in the treatment of chronic non-infectious diseases including at least three dimensions: the first is the etiological treatment, the second is the pathophysiological and pathopsychological dimension, and the third is symptomatic treatment. It shall be noticing that somatic symptoms need more attention. Psychosomatic unity and a diverse mode of clinical thinking are important to understand somatic symptoms and the treatment of somatic symptoms.

## Supporting Information

S1 File (PDF)

S2 File (DOC)

S3 File (JPG)

S4 File (JPG)
