# Supplementary material for: The Theoretical Construction of a Classification of Clinical Somatic Symptoms in Psychosomatic Medicine Theory
Source: PLoS One. 2016 Aug 15;11(8):e0161222. doi: 10.1371/journal.pone.0161222 (PMC4985123; doi:10.1371/journal.pone.0161222)
Supplement: S2 File — (DOC) [file pone.0161222.s002.doc]

Dear editors:

Thank you for your work with our manuscript.

We give some information about the time of ethics approval document in our study. The time of began recruiting participants in May 2014, the study research began on May 6,2013. We solemn commitment that the approval date was prior to began recruiting participants.

After we received the approval of ethics commitment, we began recruiting participants.

So, the time of began recruiting participants in the manuscript has been corrected.

Thank you for your patience.

Best regards,

Xueli sun, Fanmin Zeng
