# Supplementary figures and images for: The Theoretical Construction of a Classification of Clinical Somatic Symptoms in Psychosomatic Medicine Theory
Source: PLoS One. 2016 Aug 15;11(8):e0161222. doi: 10.1371/journal.pone.0161222 (PMC4985123; doi:10.1371/journal.pone.0161222)

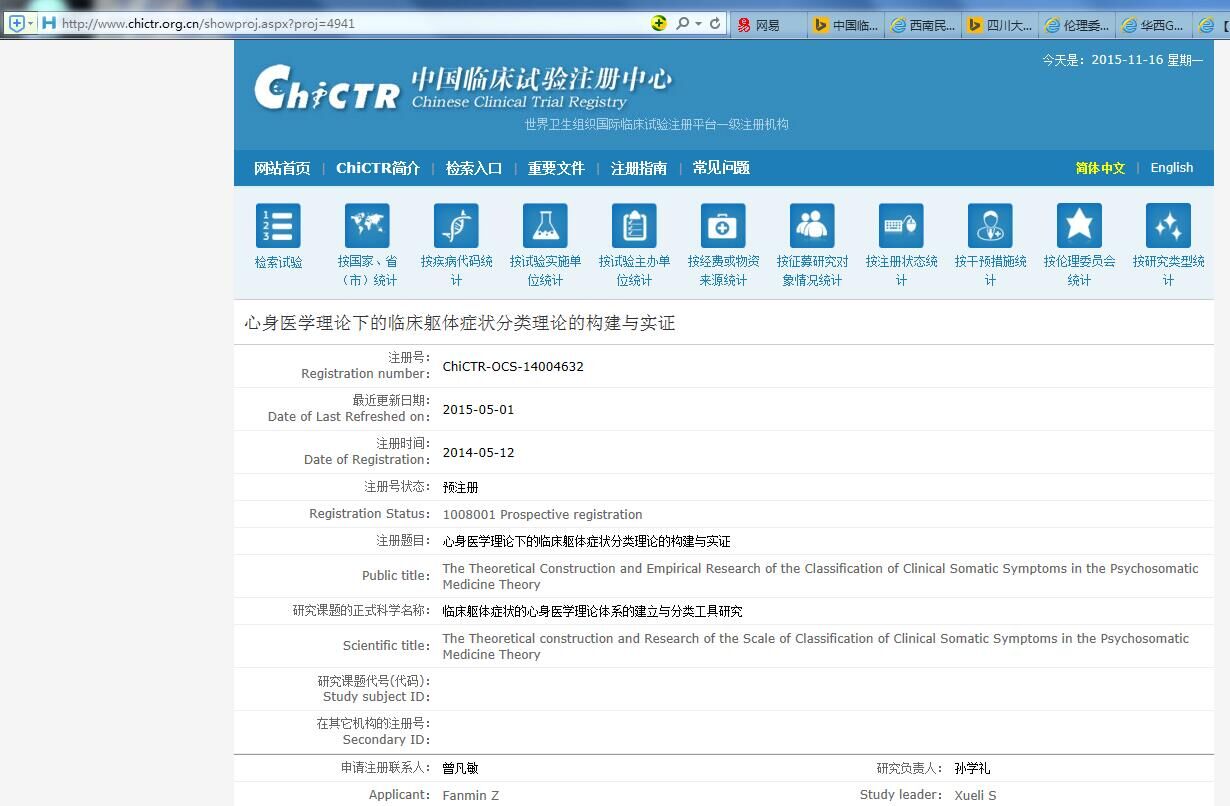

Supplement: S3 File — (JPG) [file pone.0161222.s003.jpg]

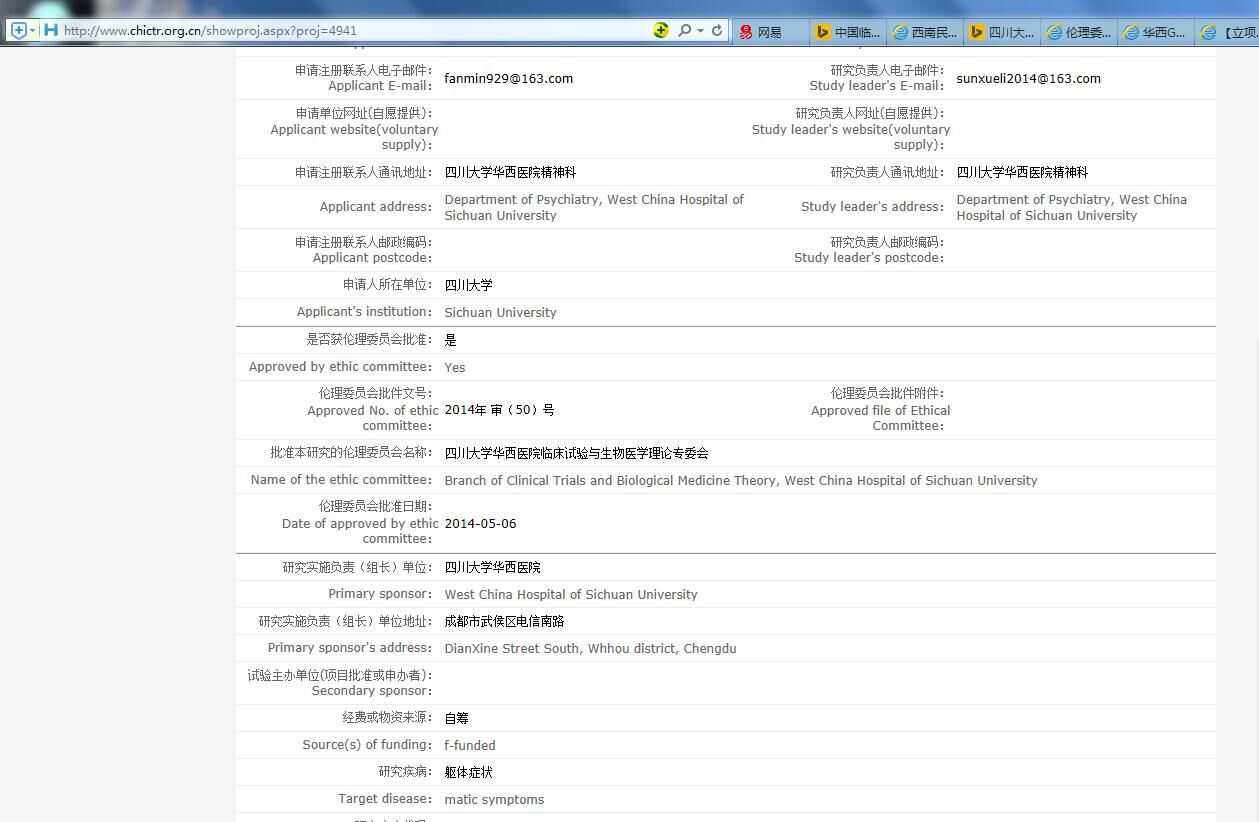

Supplement: S4 File — (JPG) [file pone.0161222.s004.jpg]
